# Quantitative gene set analysis generalized for repeated measures, confounder adjustment, and continuous covariates

**DOI:** 10.1186/s12859-015-0707-9

**Published:** 2015-08-28

**Authors:** Jacob A. Turner, Christopher R. Bolen, Derek M. Blankenship

**Affiliations:** 1 Baylor Research Institute, 3310 Live Oak, Dallas, 75204 TX USA; 2 Department of Microbiology and Immunology, Stanford University School, Stanford, 94305 CA USA

**Keywords:** Gene set analysis, Linear mixed models, Repeated measures, Confounder adjustment, QuSAGE

## Abstract

**Background:**

Gene set analysis (GSA) of gene expression data can be highly powerful when the biological signal is weak compared to other sources of variability in the data. However, many gene set analysis approaches utilize permutation tests which are not appropriate for complex study designs. For example, the correlation of subjects is broken when comparing time points within a longitudinal study. Linear mixed models provide a method to analyze longitudinal studies as well as adjust for potential confounding factors and account for sources of variability that are not of primary interest. Currently, there are no known gene set analysis approaches that fully account for these study design and analysis aspects. In order to do so, we generalize the QuSAGE gene set analysis algorithm, denoted Q-Gen, and provide the necessary estimation adjustments to incorporate linear mixed model analyses.

**Results:**

We assessed the performance of our generalized method in comparison to the original QuSAGE method in settings such as longitudinal repeated measures analysis and accounting for potential confounders. We demonstrate that the original QuSAGE method can not control for type-I error when these complexities exist. In addition to statistical appropriateness, analysis of a longitudinal influenza study suggests Q-Gen can allow for greater sensitivity when exploring a large number of gene sets.

**Conclusions:**

Q-Gen is an extension to the gene set analysis method of QuSAGE, and allows for linear mixed models to be applied appropriately within a gene set analysis framework. It provides GSA an added layer of flexibility that was not currently available. This flexibility allows for more appropriate statistical modeling of complex data structures that are inherent to many microarray study designs and can provide more sensitivity.

**Electronic supplementary material:**

The online version of this article (doi:10.1186/s12859-015-0707-9) contains supplementary material, which is available to authorized users.

## Background

Linear mixed models (LMMs) have been widely accepted as powerful approaches for modeling microarray data [1, 2]. LMMs are useful when the study designs are more complex than a traditional case-control study. For example, LMMs can be used to adjust fold change estimates between groups by accounting for confounding factors that could not be controlled through randomization [3]. This is a very important feature that can be overlooked in observational studies and can bias the fold change estimates if not adjusted properly. The linear mixed model can also be used to account for repeated measures in longitudinal studies as well as account for additional sources of variability through the inclusion of random effects in the model [1, 2]. Repeated measures can also be accounted for by modeling the residual side covariance parameters in a wide number of ways and can allow for unequally spaced time points. The flexibility and robustness of modeling options with a mixed linear model makes it a great candidate for many challenging study designs.

Another challenging aspect of many microarray analyses is detecting differential gene expression when signal is relatively weak due to technical reasons or to the specific biological components of the study. Gene set enrichment analysis can be a more powerful approach that offers statistical analyses on gene sets defined by biological function. By summarizing probe level information within a gene set, the resulting statistical inference on the gene sets often lead to very informative, statistically significant findings, even when gene level analysis produces a very small number of significant genes. The developement of gene set analysis approaches has been ongoing for the past decade. For a thorough discussion and overview of many of the gene set analysis methods see [4–8].

Our work has hinged on the necessity to incorporate gene set enrichment analysis to microarray studies with complex studies designs. Most gene set analysis algorithms were designed for simple case-control or paired study designs. Some have been extended to incorporate more general models such as one way ANOVA or linear regression models [5, 9–11]. In reality, study designs are often much more complicated due to unbalanced numbers of patient groups, missing samples, confounding factors that could not be controlled in collection, and correlated data inherent to longitudinal studies. One statistical modeling approach to tackle many of these issues is the use of general linear mixed models. The ability to model repeated measures at the probe level with either random effects or structuring the residual covariance matrix in addition to simple linear regression models is needed to obtain optimal and unbiased results. A key component to the lack of this generalization is the fact that many algorithms perform sample based permutation tests to obtain *p*-values. These permutations do not preserve the correlation structure that repeated measures have over time and thus are not appropriate to apply.

Most methods claiming to be appropriate for longitudinal studies simply will use paired t-tests when the specific comparison of interest involves paired data. Incoporating a mixed model however can provide a more stable estimate of variaibility when the number of observations at each time point is low and can specifically model subject variability. Zhang *et al* provide a nonparametric approach in GSA for longitudinal studies, but their procedure only allows for the tests of overall main and intereaction effects [12]. There is no ability to test for a specific contrast of interest over time. Our objective is to produce an appropriate gene set testing procedure that allows for the incorporation of any pairwise tests derived from a LMM.

## GSA overview and extension motivation

Goeman and Buehlman [5] provide a thorough overview of the key components of gene set analyses as well as their potential drawbacks and difficulties. We simply summarize a few key points. There are generally two types of GSA tests. Competative tests define the null hypothesis that genes within a gene set are differentially expressed as frequently as genes not within the gene set [5]. Some common competative based testing procedures include PAGE, SAFE, and the CAMERA procedures [10,11,13]. The null hypothesis of a self-contained test is that no genes are differentially expressed in the gene set. Self-contained tests are invariably more powerful than competative tests due to the fact that any null scenario under a self-contained setting is also null under the competative definition. However, the reverse is not necessarily true. This leads to a much stricter form of alternatives for the competetive test and thus a decrease in statistical power. Given this, our focus will be on self contained gene set testing.

The declaration of the null hypothesis is closely related to the resulting statistical procedure that is developed. For self contained gene set testing, a test statistic is defined to summarize the overall differential expression amongst the genes in a gene set. The null distribution of these statistics are typically deriveable based on asymptotic theory and unknown for a small number of samples. Therefore, it is quite common for the gene set algorithms to obtain appropriate *p*-values under the null by performing sample based permutations of the data. Permutating samples preserve the probe to probe correlation structure of the expression data. This is a necessary property since genes within a gene set are typically correlated with each other. Much of the debate and developement of gene set enrichment testing procedures have revolved around the selection of an appropriate and powerfull test statistic that summarizes the gene set as well as what role sample and probe based permutations should be involved in obtaining the null distribution. For example, the GSEA algorithm is a hybrid of sorts [14]. Its gene set statistic is an enrichment score based on the abundance of highly expressed genes in a gene set compared to all the other genes (competative), but obtains the null distribution of the enrichment score by permutating the sample lables (self contained). The approach developed by Efron and Tibshirani uses a maxmean statistic to quantify each gene set and they argue that permutation on both the sample and probe labels is necessary [15].

Extending a gene set analyisis methodology to incorporate complex designs such as longitudinal studies has two general requirements. The first is that statistical inference should not be based on sample permutations. We envision it is possible to achieve inference based on permutation, however, greater detail must be made to ensure that the permutations preserve the corrleation structure when there are repeated measures over time for each probe, perhaps through a decorrelate, permutate, recorrelate type procedure. Secondly, if inference is no longer based on sample permutations, the procedure must account for the correlations that exist among the probes within a gene set. Restricting ourselves to self-contained gene set testing without the use of permutation quickly reduces the field of possible contenders. It is our view that the gene set algorithm Quantitative Set Analysis for Gene Expression (QuSAGE) comes the closest to meeting our needs for extensions to linear mixed models [16].

QuSAGE approaches self-contained gene set analysis by testing whether the average log2 fold changes within a particular gene set is different from zero [16]. In addition to some of its methodological advantages, using the average log2 fold change as the gene set statistics provides easily interpritable estimates. QuSAGE’s method to test for the average fold change of a gene set is equal to zero is straight forward. Under the assumption of normality for log2 expression data, the distribution for each probe level fold change estimate is simply a shifted and scaled t-distribution. The scaling is provided by the appropriate degrees of freedom and standard error of the fold change. QuSAGE determines the distribution for the average fold change statistic by approximating the distribution of the sum of the shifted and scaled t-statistics for each probe through numeric convolution and then scales it by the size of the gene set. This allows for the calculation of confidence intervals in addition to *p*-values. An issue with the density approximation is that it assumes that each fold change estimate is independent of the other. To account for correlations among the probes within the gene set, the density is then rescaled by an estimate of the variance inflation factor (VIF) which is a common method to measure strength of correlation among multivariate variables. Inference based on the final, VIF scaled, distribution is shown to control the type-I error rate under the two indendent sample setting with unequal variances.

Another advantage to QuSAGE is post hoc testing can be conducted. A typical comparison in immunology settings is to test for differences in changes over time with respect to a baseline time point for different groups of subjects. Since QuSAGE provides a full density estimate of the average fold change for each one comparison, the density can then be used to test for the change from baseline in one group of subjects versus the change in another. This comparison is often referred to as “the difference of the differences”. However, the procedure for conducting these types of post hoc tests assumes that the two tests being compared are independent. In repeated measures designs, it is possible to compare tests that are correlated and QuSAGE’s post hoc procedure can not appropriately account for it. Fortunately, linear mixed models can directly test for these type of post hoc tests by simply writing the appropriate contrast statment. The test is directly built into the probe level analysis so that no post hoc testing is then required.

The QuSAGE method is appealing for the incorporation of more general models used in longitudinal studies due to the fact that, with exception of the VIF estimation, all that is needed to provide statistical inference is the information used in the construction of the gene level t-statistics. At first glance, it might seem reasonable to simply feed results from any statistical model the user chooses through the QuSAGE algorithm and estimate the VIFs using the functions provided within QuSAGE’s Bioconductor package. Through our investigation of this approach, it is clear that in order to incorporate the QuSAGE method to a more general framework, care must be taken with the VIF estimations in particular when additional sources of variablity from continuous covariates or random effects are present. If the VIFs are incorrectly estimated, type-I error rates can be drasticaly inflated or deflated depending on the setting. This could lead to erroneous results. Our extension to QuSAGE adjusts the VIF estimation procedure so that the QuSAGE methodology can be applied with models typically used in longitudinal settings that appropriately control the type-I error.

### Our approach

1. Fit LMM appropriate for study design and obtain the t-statistic information required by QuSAGE
2. Obtain the conditional residual matrix of the gene expression data derived from the LMM
3. If a random effect is present and has few number of observations per level, refit the model treating the random effect as fixed and obtain the residual matrix
4. Calculate VIFs on final residual matrix assuming equal or unequal variances as specified by the LMM
5. Run QuSAGE methodology with model specific fold changes, standard errors, and adjusted VIFs

## Methods

The methodology we present can be applied to any general linear mixed model. Following the notation of the QuSAGE authors, let *E*_*i*_ represent the *i* ^*th*^ probe in a gene set of interest that is an *n* x 1 vector of expressions where *n* is the total number of samples. The linear mixed model for a single probe can be written in matrix form as

$$E_{i}=X\beta_{i}+Z\gamma_{i}+\epsilon_{i} $$

where *X* and *Z* are design matrices for fixed and random effects respectively, *β*_*i*_ is a vector of fixed effect parameters, *γ*_*i*_ is a multivariate normal random effects vector with mean 0 and covariance matrix *Σ*_*rand*_, and *ε*_*i*_ is a multivariate normal random vector of residuals with mean 0 and covariance matrix *Σ*_*res*_. Note the model above covers a wide variety of scenarios including all of the models discussed in the original QuSAGE paper. Statistical inference can then be applied for various hypotheses by testing different contrasts of *β* using general t-statistics with appropriate standard errors while accounting for repeated measures, potential confounders, and additional sources of variability.

Let *F* *C*_*i*_, *s*_*i*_, *d* *f*_*i*_, be the corresponding fold change estimate, standard error, and degrees of fredom used in construction of test statistics for a particular hypothesis for probe *i*. Applying the QuSAGE method to obtain a full probability density for the average fold change of a gene set can now be obtained under the assumption of uncorrelated probes. To adjust for the correlations present between probes in a gene set, the density must be scaled by a variance inflation factor. The QuSAGE authors propose two methods to estimate the VIF, one assuming equal variances among the conditions being modeled within the data set and the other assuming unequal. We will introduce the equal variances estimation first as it provides a reasonable transition into a more generalizeable estimation technique.

For a set of *N* genes, *E*_1_,…,*E*_*N*_, the typical VIF estimate is defined by

(1) $$ \widehat{VIF}=\frac{\sum\limits_{i=1}^{N} \sum\limits_{j=1}^{N} \widehat{Cov}(E_{i},E_{j})}{\sum\limits_{i=1}^{N} \widehat{Cov}(E_{i},E_{i})}   $$

where $\widehat {Cov}$ is the standard covariance estimate between two normal random variables. This simple formula is valid when the samples from the expression set are independent and have a common mean and variance. Since typical expression sets have samples coming from more than one condition it is more than likely that the samples do not share a common mean. To account for this the QuSAGE authors, define the covariance estimates on a group of samples indexed by *g* where *g*∈*G* are the indexes of the samples that belong to a single group and G is one of the conditions, typically either control or treatement. The covariance estimate of group *G* is defined as

(2) $$ \widehat{Cov_{G}}(E_{i},E_{j})=\frac{\sum\limits_{g \in G} \left({E^{g}_{i}}- \bar{{E^{G}_{i}}}\right)\cdot\left({E^{g}_{j}}- \bar{{E^{G}_{j}}}\right)}{N_{G}-1}   $$

where $\bar {{E^{G}_{i}}}$ is the usual sample mean for group *G* and *N*_*G*_ is the total number of samples within group *G*. If one assumes equal covariances across the conditions in the expression set, the individual group covariance estimates can be pooled together to estimate the overall variance

(3) $$ \widehat{Cov_{p}}(E_{i},E_{j})=\frac{\sum\limits_{G \in {T,C}} (N_{G}-1) \cdot \widehat{Cov_{G}}(E_{i},E_{j}) }{\sum\limits_{G \in {T,C}} (N_{G}-1)}   $$

With the assumption of equal variances, the covariance estimate in Eq. 1 can be replaced with that of the pooled estimate in Eq. 3 and the estimated VIF is updated and accounting for the additional variability due to the conditions of the samples. If it is assumed that the variances are not equal, then the VIF is estimated separately for each group using Eq. 2 and averaged together and weighted by the number of samples in each group.

A more general VIF estimation technique can intuitively be argued by the numerator in Eq. 2. Regardless of the index, the component ${E^{g}_{i}}- \bar {{E^{G}_{i}}}$ used to calculate the covariance is simply the residual of fitting a linear model with a single main effect for the condition. The residual has variability due to the condition subtracted out so the VIFs are estimated using the variability due to measurement error. The VIF estimate is exactly the same whether you perform QuSAGE’s technique on the raw data or you conduct the VIF estimates on a raw residual expression matrix. We explicitly use the term raw residual advocating that no other transformation or standardization of the residuals need to be calculated which is inherent to other VIF estimation techniques such as the method implemented in the competitive gene set approach CAMERA [11]. The reason for this is that the VIF is not invariant to transformations. For example, the VIF of a particular covariance matrix will not equal the VIF of its corresponding correlation matrix, which is just a simple standardization of the variables to have mean zero and unit variance. We have found that using standardized residuals provides more conservative results.

QuSAGE only allows the VIF estimate to account for categorical conditions in the data that are potential sources of variaibility, however, for the general linear mixed model, additional sources of variaibility can be present due to continuous covariates and random effects. The VIF estimates can not fully be realized under the general framework of QuSAGE and are potentially biased. For the general linear model setting, one simply needs to obtain the VIF estimates using the residual expression values of the model, rather than the raw expression. For the equal variance approach, our general pooled (gp) covariance estimate used for VIF calculation is

(4) $$ \widehat{Cov_{gp}}\left(E_{i},E_{j}\right)=\frac{\sum\limits_{k=1}^{n} \left({E^{k}_{i}}- \hat{{E^{k}_{i}}}\right)\cdot\left({E^{k}_{j}}- \hat{{E^{k}_{j}}}\right)}{n-p}  $$

where ${E^{k}_{i}}$ is the *k* ^*th*^ sample expression value for gene *i* and $\hat {{E^{k}_{i}}}=X\hat {\beta _{i}}+Z\hat {\gamma _{i}}$, the Best Linear Unbiased Predictor (BLUP) from the linear model of gene *i*. The residuals defined in this way have all sources of variability due to both fixed and random effects removed. If the linear model allows for unequal variance estimation across a particular group of categorical conditions, then the original QuSAGE technique can be applied to the residual matrix of expression values to account for the unequal variances. This procedure is valid for all linear models and linear mixed models when the random effects have an adequate number of observations per random effect level. For longitudinal microarray studies where the individual subject is used as a random effect, we suggest that four time points is adequate. When the number of observations is low, the conditional residuals from the mixed model are shrunk closer to zero, biasing the VIF estimate. The shrinkage of the residual is inherant to linear modeling with random effects [17]. To correct for this, residuals must be obtained from a sperate linear model that treats the random effects as fixed effects.

In summary, our generalization of the QuSAGE method, denoted as Q-Gen, allows for one of the most flexible and general modeling techniques, the linear mixed model, to be incorporated in a gene set analysis approach. By incorporating statistical models appropriate to the study design, adjusted fold change and variability estimates within QuSAGE, along with their associated *p*-values are more reliable. Effects of ignoring statistical issues such as confounders and random effects can lead to erroneous results.

## Results and discussion

### Simulation studies

We use simulation studies to illustrate the advantages and necessity of using residuals to estimate the VIF when incorporating the QuSAGE method with probe level analysis derived from LMMs. For conciseness, we compared QuSAGE to Q-Gen when analysing a single gene set. The simulation study consists of two main components, one involving the inclusion of a confounding variable. The second component is to explore the effect of repeated measure designs and longitudinal studies.

The simulations were generated using the following procedure. First, a residual matrix of expression values, *R*_*pxn*_, where *p* is the number of genes in the gene set and *n* is the number of samples, is randomly generated from a multivariate normal distribution with a defined covariance structure so the VIF is known. A signal matrix *S*_*pxn*_ is then generated row by row from the linear mixed model framework *X* *β*+*Z* *γ*, where *β* and *γ* are the specified fixed and random effects for that gene. The final simulated expression data set was then obtained by simply adding *E*=*S*+*R*. QuSAGE and Q-Gen were then applied on 10,000 simulations and the estimates of the type-I error rates, VIFs, and power were recorded and compared across a number of scenarios including varying degress of VIFs. We considered scenarios in which the genes within the gene set were uncorrleated, pairwise correlated 0.2, and pairwise correlated 0.7 and correspond to VIFs of 1, 6.434, 20.024 respectively.

For our first study, we compared the effects of a simple case-control study, 5 and 15 samples within each group, with the addition of a confounded continuous covariate variable, age, that can be described by the following linear model

(5) $$ E=\beta_{0}+\beta_{1}X_{treat}+\beta_{2}X_{age}+\epsilon  $$

where *β*_0_ is the control level mean expression set to 6, *β*_1_ is the added effected of treatment group. *X*_*age*_ is a continuous covariate we selected that ranges from 15 to 30 with a mean of 25 to mimic a clinical variable such as age and its regression coefficient *β*_2_ was set to 0.03. This can be interpreted as an increase of a subject’s age by 10 years corresponds to an increase in expression level of 0.3. In addition, we confounded the variable with the treatment group so that larger values of *X*_*age*_ were primarily contained in the treatment group. Setting *β*_1_=0, we assessed the type-I error rates and VIF estimates of the original QuSAGE method and Q-Gen using a mixed model adjusted for *X*_*age*_. In addition to varying degrees of VIFs, we also considered the abundance of genes within a gene set that truly had the confounder present. We considered three cases. We looked at a control scenario when no probes have a confounder, 0 % as well as when 25 and 50 % of the probes have the confounding variable present within its expression.

Table 1 provides a summary of both VIF estimates and type-I error rates. Under the control scenario of no confounding present, there is relatively no difference. They both adequately estimate the VIF and control type-I error rate at the 0.05 level. As more and more probes are included with the confounder, the VIF and the fold change estimates of the original QuSAGE method become biased and the type-I error rates are inaccurate. An interesting point for the larger sample size scenario is that when the true VIF is equal to one, the VIF is overly estimated suggesting that the test would be more conservative, but in somewhat contradictory fashion, still has an inflated type-I error rate. This is due to the fact that the fold change estimates for QuSAGE are biased and even though the over estimated VIF is providing a harsher penalty, the magnitude of the bias in the fold change estimate is overwhelming. This was not seen in the small sample size scenario because the degree of confounding was not as extreme.

**Table 1:** VIF and Type-I Error Control in the presence of a confounder

			VIF Estimate		Type-I Error	
Sample Size	Percent Conf.	True VIF	QuSAGE	Q-Gen	Qusage	Q-Gen
5	0	20.024	19.04	18.89	**.046**	**.054**
5	.25	20.024	17.43	18.87	.042	.050
5	.5	20.024	17.89	18.89	.041	.055
5	0	6.435	6.20	6.16	.044	**.050**
5	.25	6.435	6.282	6.2	.040	.054
5	.5	6.435	8.04	6.16	.045	.055
5	0	1	0.99	0.99	**.048**	**.053**
5	.25	1	1.72	1.00	.041	.054
5	.5	1	4.16	0.99	.010	.057
15	0	20.024	19.75	19.74	**.047**	**.047**
15	.25	20.024	18.72	19.72	.056	.049
15	.5	20.024	18.59	19.71	.0822	.053
15	0	6.435	6.36	6.36	**.053**	**.052**
15	.25	6.435	6.43	6.36	.072	.052
15	.5	6.435	8.27	6.34	.101	**.051**
15	0	1	1.00	1.00	**.048**	**.049**
15	.25	1	1.719	1.00	.097	.050
15	.5	1	4.176	1.00	.074	**.051**

VIF and type-I error estimates under a case-control simulation in the presence of a confounding variable where the percentage of genes that are affected by the confounder are examined for 0, 25, and 50 % respectively. Error rates in bold indicate they are within the margin of error (0.05 ± 0.00427) for the simulation study. Since Q-Gen allows for a linear model that adjusts for covariates, VIF estimation and the controlling of the type-I error is more consistent than the standard two sample t-testing conducted within QuSAGE

**Fig. 1:**
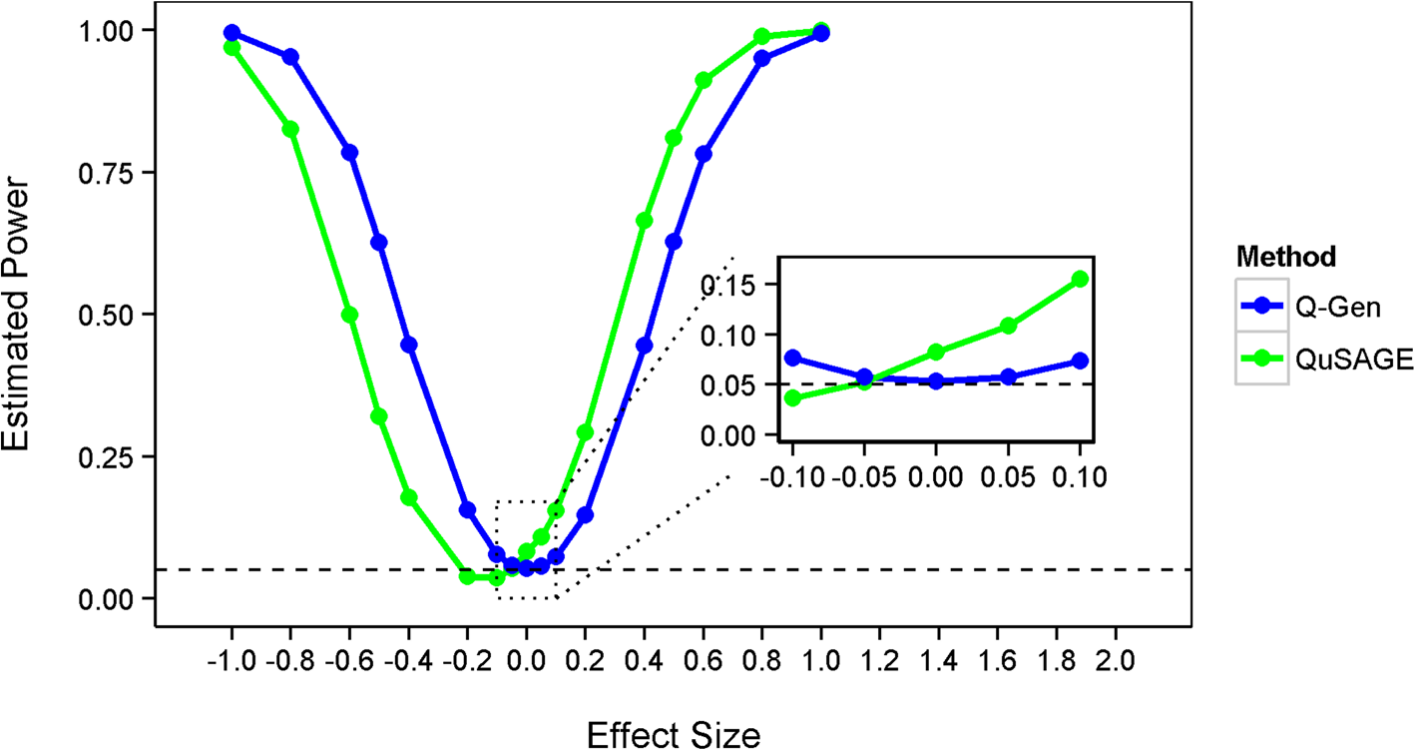
Power estimates in the presence of a confounder. Plot of estimated power vs. true fold change differences (effect size) using a significance threshold of 0.05 under a gene set simulation with *V* *I* *F*=20.024 and 50 % of probes are confounded. QuSAGE’s power curve is biased to the left and does not maintain a minimum at zero. Q-Gen’s adjustments appropriately controls for type-I error rate and its statistical inference can be trusted when true differences occur

The effect of the confounding variable can be illustrated further by examing the properties of the power curves of the two methods. Figure 1, provides the power estimates when 50 % of the probes are confounded and the VIF is equal to 20.024 under the 15 samples per group scenario. It is clear that the entire power curve is biased and shifted to the left which creates the inflation of the type-I error rate. Since the confounder slightly increases the expression within the treatment group, it is much harder to detect a down regulated gene set. The conservativeness of QuSAGE’s VIF estimate can be seen where its minimum power estimate is below 0.05.

In our second simulation, we investigated the effects when an additional source of variability is present in the expression other than measurement error in the residual. In a longitudinal study when subjects have repeated measurements over time, a random effect can be used to take into account for subject specific variability. Under this particular model, correlation between the repeated measures over time is assumed to be equal between any two pairs of time points [18]. Using the same VIF values, we simulated longitudindal data sets of 5 and 15 subjects having 2, 5, and 10 repeated measures. For each number of repeated measures, we investigated the properties of testing the difference between the second and first replicate, or in other words, difference between the first time point and a baseline timepoint. This should have no consequence on QuSAGE’s paired t-test approach, but the added replicates will help the linear mixed model estimate the random effect which could have an influence. The parameters for the subject specific random effect were chosen such that the pairwise correlation between any two time points is 0.7.

**Fig. 2:**
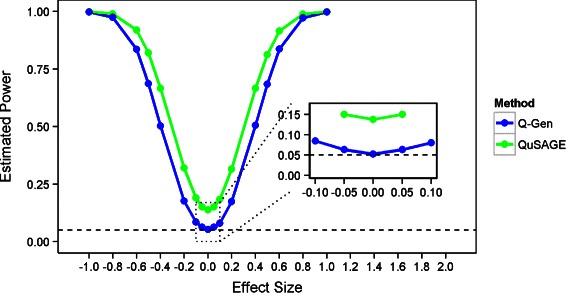
Power estimates in the presence of a random effect. Plot of estimated power vs. true fold change differences (effect size) using a significance threshold of 0.05 under a longitudinal gene set simulation (*V* *I* *F*=20.024) with five time points using a random effect for subject. The underestimation of the VIF under QuSAGE yields overly optimistic *p*-values. Q-Gen remains conservative for an adequate number of replicates

Table 2 provides the simulation results under the random effects model setting. The QuSAGE method across all scenarios drastically underestimates the VIF, and thus inflates the type-I error rate. Q-Gen adequately controls the type-I error rate across most of the scenarios and exhibits mild inflation when the number of subject replicates is low. Examining the power curve in Fig. 2 for five replicates on 15 subjects and a VIF of 20.024, the fold change estimates are unbiased for both estimates as both methods obtain a minimum when there is no change between the two time points. However, there is an upward shift in the power curve for QuSAGE indicating it’s over optimism due to the under estimation of the VIF.

**Table 2:** VIF and Type-I Error Control in the presence of a random effect

			VIF Estimate		Type-I Error	
Sample size	Replicates	True VIF	QuSAGE	Q-Gen	Qusage	Q-Gen
5	2	20.024	10.02	18.08	.129	**.049**
5	5	20.024	10.38	18.47	.114	**.052**
5	10	20.024	10.44	19.46	.105	**.054**
5	2	6.435	3.63	5.99	.093	.055
5	5	6.435	3.69	5.99	.077	.055
5	10	6.435	3.70	6.28	.061	**.048**
15	2	20.024	10.37	19.44	.147	**.052**
15	5	20.024	10.47	19.13	.138	**.052**
15	10	20.024	10.49	19.75	.137	**.051**
15	2	6.435	3.69	6.27	.124	.057
15	5	6.435	3.72	6.18	.121	**.054**
15	10	6.435	3.71	6.38	.114	**.053**

VIF and type-I error estimates under a longitudinal simulation in the presence of a subject specific random effect. Under the current simulation setting, the VIF estimates under QuSAGE are drastically underestimated and lead to inflated type-I error rates. Error rates in bold indicate they are within the margin of error (0.05 ± 0.00427) for the simulation study. The type-I error rates for Q-Gen are consistantly in control with mild error inflation when there are fewer replicates

The reason for this drastic difference in VIF estimates is because the subject specific variability was added to each probe independently in our simulations. Random effects are random variables just like the residual component of a linear model, when QuSAGE estimates the VIF, the independence of the variablity between subjects and the correlation between the probes cannot be parsed out. Therefore, the QuSAGE estimate is pulled closer to a VIF of one. This of course may not be realistic in real data, but the only way for the VIF estimates from QuSAGE and Q-Gen to correspond exactly under this scenario is when the covariances of the random effects and the covariance of the probes are exactly the same. We do not feel this is a safe assumption in reality, and recommend the Q-Gen approach as the residuals have the covariances due to the random effects subtracted out.

Another approach to modeling longitudinal expression data with linear models is through structuring subject specific correlations in the residuals. For example, autoregressive properties that assume the correlations between time points decay exponentially the farther apart they are in time. We explored the aspect of AR(1) processes in a similar fashion as our random effects simulation. Although we do not provide the results, the VIF estimates are identical and the only difference that can be seen is in the slightly more powerful linear model. One must also consider that in real data, adjusting for confounders, adding random effects, and specifying residual side covariance structures could be called for in one single model. We only consider the effects of each one of these components one at a time in our simulations. Thus, the reasons why QuSAGE and Q-Gen may differ in real analysis settings would be harder to assess. From these simulation studies, it is clear that adding additional complexities inherent to longitudinal and observational study designs can have a negative impact on the original QuSAGE methodology. Our generalization provides a frame work to overcome these issues.

### Influenza study revisited

We re-examined the influenza study originally presented in the QuSAGE paper [19]. Data from this study is publically available and reported by the researchers to have been “approved by the relevant institutional review boards and conducted according to the Declaration of Helsinki.” The normalized expression data was downloaded from the GEO database (GEOID: GSE30550). In this study, temporal whole blood gene expression data was taken from 17 healthy human subjects before and after they were challenged with the H3N2 live influenza virus. After an initial baseline measurement, 14 additional time points up to 108 hours post challenge were collected at unequally spaced time intervals. Each subject was identified as either being symptomatic or asymptomatic to the challenge. The QuSAGE authors illustrated how QuSAGE could identify changes in interferon related gene sets with respect to baseline measurements earlier in time than previous GSA methods such as GSEA and CAMERA within the symptomatic subjects. However, if a single gene set analysis would survive any multiple testing correction procedures when an analysis is highly exploratory and a large number of gene sets are used was not explored.

To illustrate the advantages of incorporating a linear mixed model inside of the QuSAGE frame work, we compared Qusage analysis to our generalized approach Q-gen using 260 immunologically derived gene sets, three of which are annotated as interferon gene sets [20]. The linear mixed model used for Q-Gen included time, subject condition, their interaction term, a subject specific random effect to account for the repeated measures over time, and additional covariates to adjust the analysis for age and gender. An assumption for equal variances was also made. Tests for changes over time within condition groups as well as changes between condition at each time point were conducted using contrast statements from the linear mixed model and *p*-values were adjusted using the Benjamini-Hochberg procedure (FDR) for each comparison.

The grouping of asymptomatic and symptomatic subjects was conducted while the study was ongoing and cannot completely control for potential confounders. For example, although all the subjects could be considered similar in age (median 25.5), there is not a way to guarantee that patients within asymptomatic and symptomatic conditions will have a similar distribution. The subjects mean age by condition are almost identical, 27.5 and 27.1 respectively. However, seven out of the eight symptomatic subjects are 25 or older while five out of the eight asymptomatic subjects are less than 25 years old. Therefore, there is potential for an age confounder effect within the expression data.

**Fig. 3:**
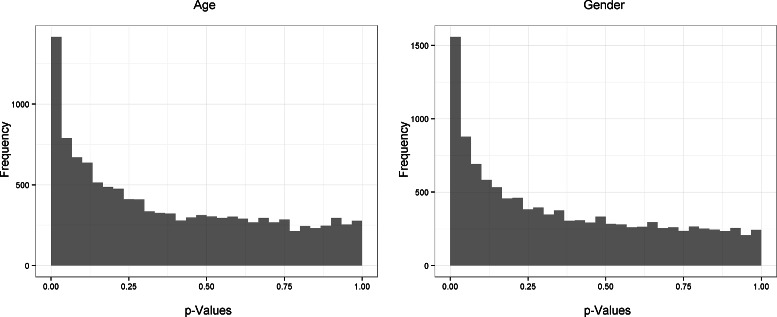
Age and gender effects within the flu longitudinal study. Distribution of *p*-values from testing age and gender effects under the linear mixed model at the gene level

The distibution of *p*-values obtained from testing the effects of age and gender at the gene level are presented in Fig. 3. Both distributions are highly positively skewed indicating a significant number of genes contain variation due to these variables that are ignored in the original QuSAGE framework. Due to theses effects in addition to subject variablity, the VIF estimation technique of QuSAGE is suspect.

**Fig. 4:**
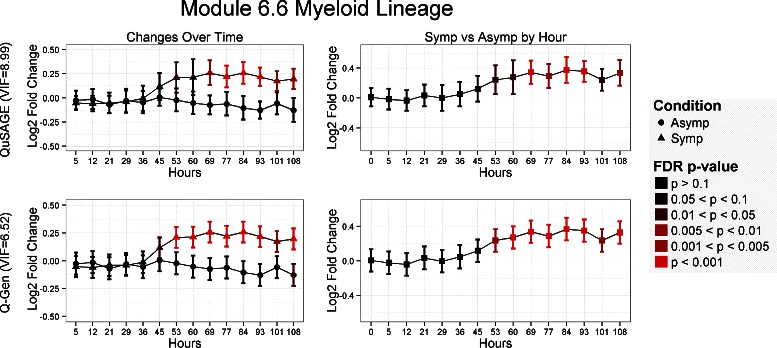
Comparison of QuSAGE and Q-Gen analysis for the M6.6 myeloid lineage gene set. Results are organized as follows: QuSAGE across the top, Q-Gen across the bottom. The left column provides the fold change estimates of testing changes over time relative to baseline, and the right column for testing symptomatic versus asymptomatic at each time point. Each comparison’s significance level is color coded based on its FDR adjusted p-value for that particular comparison across all 260 gene sets. Q-Gen’s VIF estimation is less conservative and, combined with the pooled estimate of the variance, provides narrower confidence intervals and provides additional sensitivity at earlier time points

**Fig. 5:**
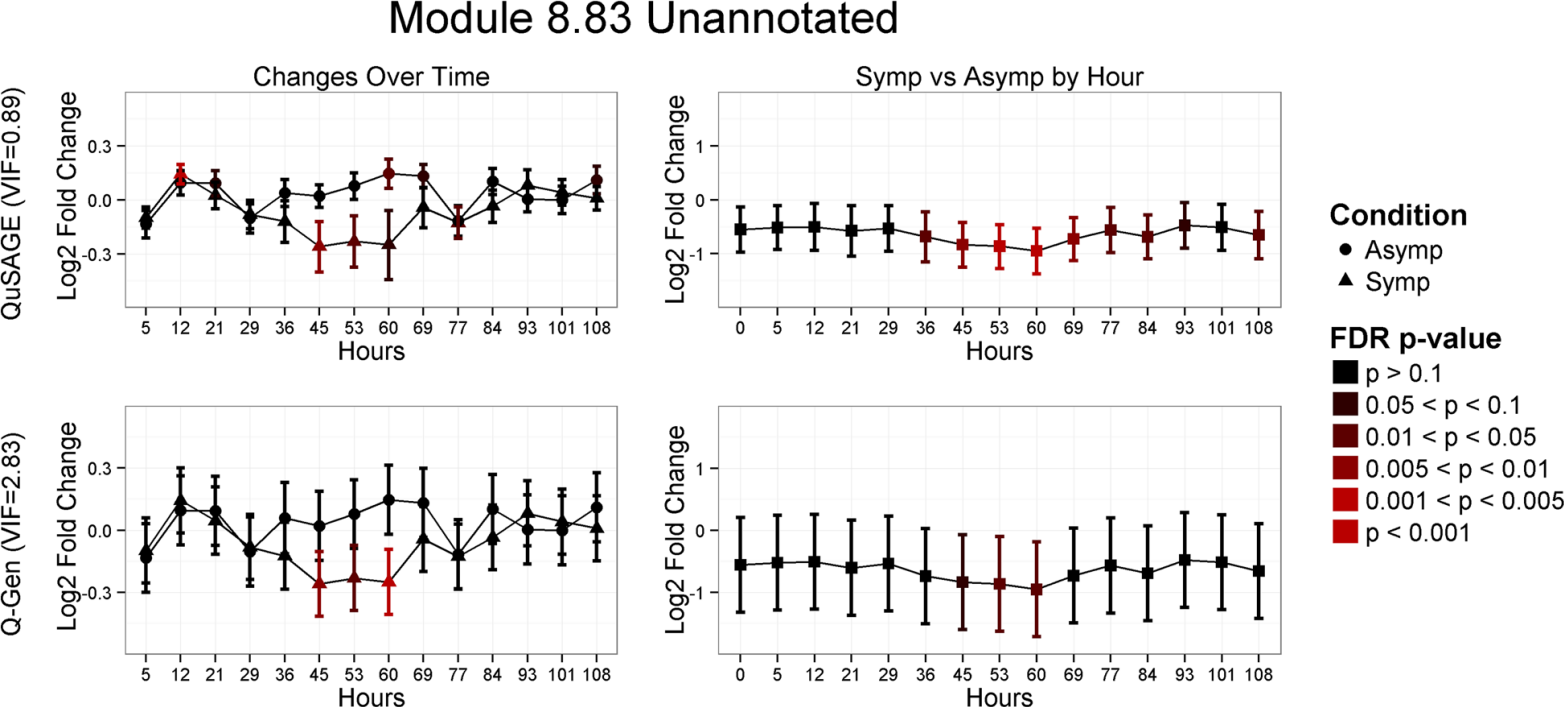
Comparison of QuSAGE and Q-Gen analysis for the M8.83 unannotated gene set. Results are organized as follows: QuSAGE across the top, Q-Gen across the bottom. The left column provides the fold change estimates of testing changes over time relative to baseline, and the right column for testing symptomatic versus asymptomatic at each time point. Each comparison’s significance level is color coded based on its FDR adjusted p-value for that particular comparison across all 260 gene sets. Q-Gen’s VIF estimation is more conservative at almost three times that of QuSAGE. This yields wider confidence intervals and more conservative *p*-values across all of the comparisons under Q-Gen

Figures 4 and 5 illustrates by example how the different estimates in VIF can have an impact on analyses. Gene set module M6.6 in Fig. 4, which is annotated as a myeloid lineage, provides an example when QuSAGE VIFs can be too conservative. QuSAGE’s VIF estimate is 8.99 versus Q-Gen’s estimate of only 6.52. This discrepancy is highlighted by the testing of changes over time with respect to baseline as the confidence intervals for QuSAGE are consistently larger than that of Q-Gen. Changes over time and changes between symptomatic and asymptomatic groups are detected up to two or three timepoints earlier with Q-Gen after FDR correction.

Gene set module M8.83 is an unannotated gene set but provides one of the biggest discrepancies in VIF estimates between the two methods. Q-Gen estimates the VIF to be 2.83 which is over three fold higher than QuSAGE’s estimate of 0.89. The confidence intervals provided by Q-Gen are drastically wider than that of QuSAGE as seen in Fig. 5. This leads to more conservative results for this particular gene set.

**Fig. 6:**
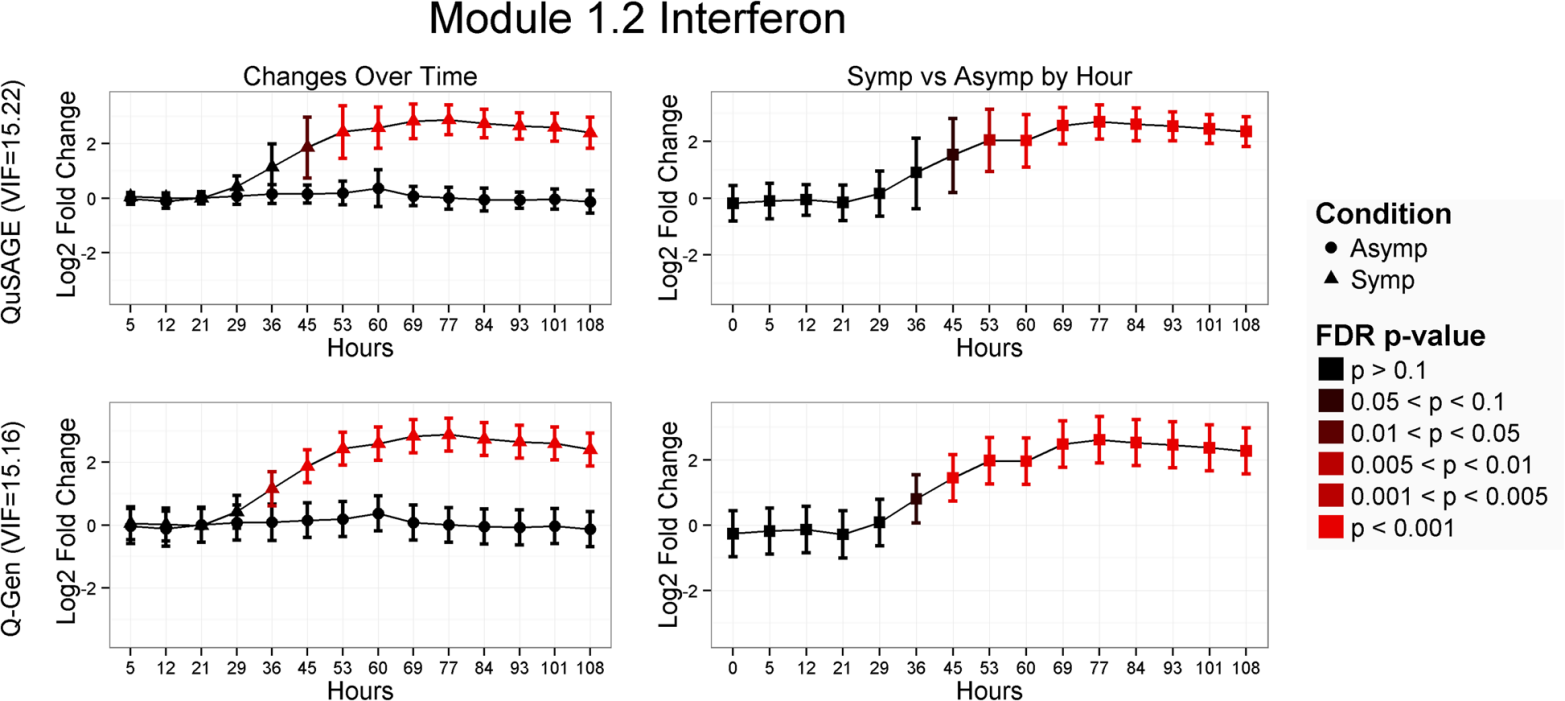
Comparison of QuSAGE and Q-Gen analyses for the M1.2 Interferon gene set. Results are organized as follows: QuSAGE across the top, Q-Gen across the bottom. The left column provides the fold change estimates of testing changes over time relative to baseline, and the right column for testing symptomatic versus asymptomatic groups at each time point. Each comparison’s significance level is color coded based on its FDR adjusted p-value for that particular comparison across all 260 gene sets. Here, both method’s VIF are similar and thus provides a good comparison of just the difference between simple t-test procedures versus a linear mixed model. Q-Gen is able to detect differences over time and between groups a few time points earlier

Figure 6 displays a comparison of results between QuSAGE and Q-Gen for gene set module M1.2 which is annotated as interferon. The collection of genes is mostly induced by type I and type II interferon. Twenty one out of the twenty seven genes are identified as being IFN-induced. The VIF factors between the two methods are very comparable as are the estimates and confidence intervals. However, the additional power the linear mixed model provides by pooling the variance and accounting for the variability contributed to age and gender is apparent across all genes and all gene sets. The real advantage of this added sensitivity is the ability to detect the earlier changes at hours 36 and 45 even after multiple testing correction was conducted. Similar findings can be found with the remaining two interferon gene set modules M3.4 and M5.12.

In this particular study, the number of subjects for the asymptomatic and symptomatic groups is eight and nine respectively. We assessed the sensitivity of Q-Gen compared to the original QuSAGE method when the number of subjects is even lower. We reduced the original analysis to include only the symptomatic group and investigated the results for comparing hour 60 versus baseline. Using a raw p-value threshold of 0.05, the total number of significant gene sets for Q-Gen and QuSAGE are 138 and 66 respectively. We then conducted a simulation using the raw data to assess the effects of reducing the number of subjects.

**Fig. 7:**
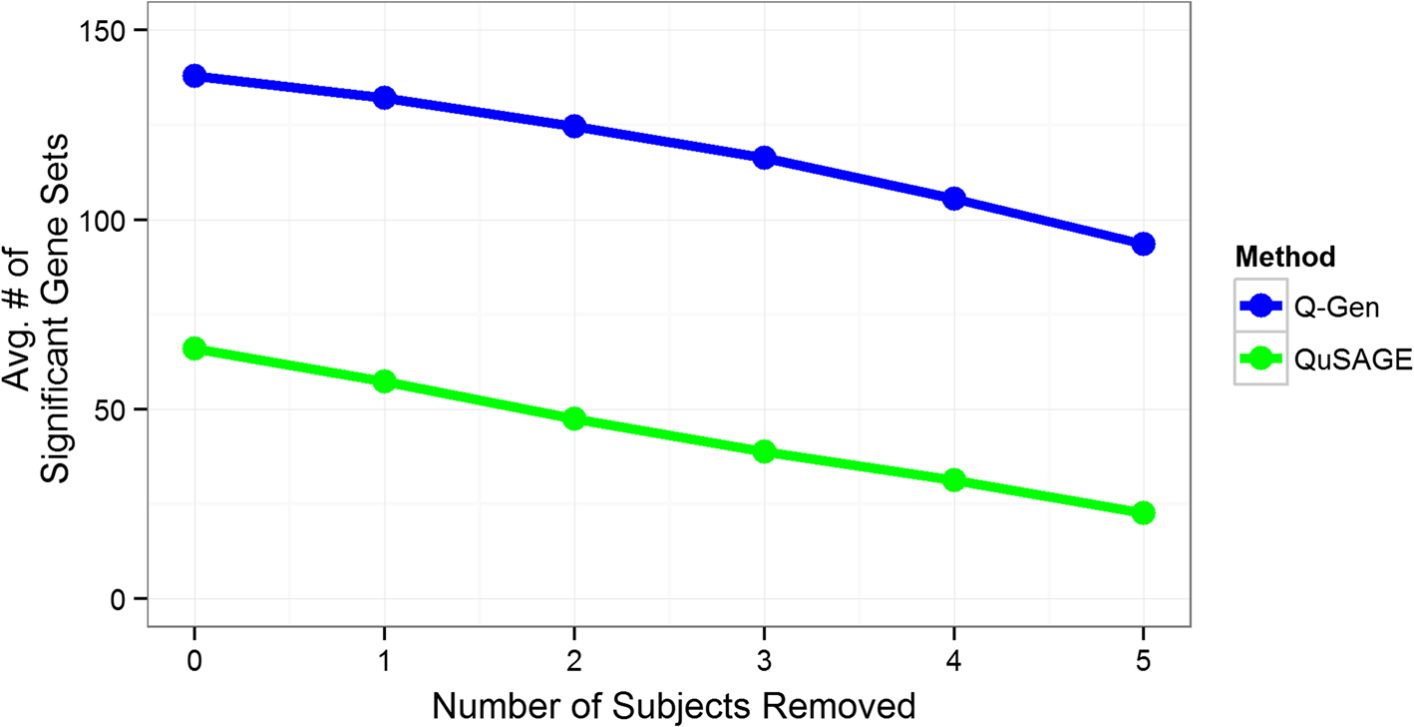
Comparison of QuSAGE and Q-Gen analyses when reducing the total number of subjects. Plot of the average number of significant gene sets as a function of the total number of subjects removed when making the comparison of hour 60 versus baseline within the symptomatic group. The averages are calculated by conducting QuSAGE and Q-Gen analysis on all data set combinations of removing the specified number of subjects indicated on the horizontal axis. Q-Gen provides higher averages consistently while both methods decline at a similar rate

The simulation was conducted by performing Q-Gen and QuSAGE on every possible data set with removing just one subject. Since there were nine original subjects, there were nine different data sets of removing one subject. We observed the total number of gene sets that were statistically significant at the 0.05 significance level for each data set and calculated the average number of significant gene sets. We repeated the process for removing two, three, four, and five subjects. Figure 7 plots the average number of significant gene sets as a function of the number of subjects removed. The plot illustrates one of the main advantages linear mixed models can have in longitudinal studies as it can take advantage of the many replicates and produces higher number of significant gene sets consistently even as the number of subjects decrease.

## Limitations

The combination of linear mixed model analysis and QuSAGE makes for a flexible and powerful gene set enrichment approach. However, there are limitations. Specifically, the limitations of linear mixed models, and statistical modeling in general, are inherently limitations of Q-Gen [21]. For example, the accuracy and precision of the covariate data is positively associated with the accuracy and precision of estimates obtained from the model. In other words, when the data is inaccurate and/or imprecise, the results will be as well. Also, covariates are modeled with an assumed association with the outcome, linear in most cases, which may be inaccurate. Correlated covariates, also known as multicollinearity, will cause problems estimating the model parameters and standard errors. The diagnostics for these issues are difficult to assess across tens-of-thousands of models run for an expression data set. Additionally, whether adjusting for confounders by including them in the model directly or via other methods such as propensity scores, biased results is still a possibility due to unknown confounders not included in the analysis. Lastly, sample size relative to the number of parameters estimated in the model should be considered. Sample size is study specific, but, as a rule of thumb, there should be at least ten observations per covariate included in the model [22].

## Conclusions

Q-gen is a gene set analysis method which extends the current QuSAGE methodology to more flexible linear mixed models that can account for confounding variables and random effects that are often used to model the repeated nature of longitudinal studies. Although the original QuSAGE method can be more powerful than other gene set approaches such as GSEA and CAMERA by more appropriately accounting for the intergene correlation of the genes within the gene set, the VIF estimation technqiue can not appropriately estimate the VIF when confounding factors and random effects exist in the data. Fortunately, accounting for this issue is corrected through the methods presented in this paper and the wonderful tools and interpretations provided by the original QuSAGE package in Bioconductor can still be used to its fullest. We provide Q-Gen as an R function along with some additional documentation and example code which is available in the Additional files 1 and 2. An implementation of the Q-gen methodology will be made available through the QuSAGE package in the next update of Bioconductor in October, 2015.

## Additional files

Additional file 1
**An R script which provides a user made function to implement the Q-Gen methodology.** The original QuSAGE package is all that is needed. Example code of how to implement the function is provided as well. (TXT 2.61 KB)

Additional file 2
**Documentation similar to that of an R help file defining the requirements of the Q-Gen function inputs and details.** (DOCX 14.2 KB)

## Abbreviations

GSA: Gene set analysis
LMM: Linear mixed models
QuSAGE: Quantitative set analysis for gene expression
Q-Gen: Generalized QuSAGE
VIF: Variance Inflation Factor
FDR: Benjamini-Hochberg false discovery rate
