# Supplementary material for: Quantitative gene set analysis generalized for repeated measures, confounder adjustment, and continuous covariates
Source: BMC Bioinformatics. 2015 Aug 28;16:272. doi: 10.1186/s12859-015-0707-9 (PMC4551517; doi:10.1186/s12859-015-0707-9)
Supplement: Additional file 2 — Documentation similar to that of an R help file defining the requirements of the Q-Gen function inputs and details. (DOCX 14.2 KB) [file 12859_2015_707_MOESM2_ESM.docx]

Generalized qusage of differential gene expression results from a linear mixed model

**Description**

A wrapper function that implements the qusage algorithm on gene expression results derived from any linear mixed model

**Usage**

qusage_gen(resids, labels, estimates, dof, std.errors, geneSets, var.equal=TRUE)

**Arguments**

resids An ExpressionSet or matrix containing the corresponding raw residuals obtained from a linear mixed model applied to each row of log2 normalized expression data.

labels Vector of labels representing the groupings of the column of resids.

estimates Numeric vector of log2 fold change estimates from a single comparison obtained from the linear mixed model. Should correspond to the order in which the genes are present in resids.

dof Numeric vector of the degrees of freedom associated with the t-statistic for the corresponding comparison of estimates. Should correspond to the order in which the genes are present in resids.

std.errors Numeric vector of the standard errors associated with the t-statistic for the corresponding comparison estimates. Should correspond to the order in which the gene are present in resids

geneSets Either a list of pathways, or a single vector of gene names corresponding to a single gene set

var.equal Specifying if the linear mixed model assumed equal variances or not. If F, the labels input should correspond to the groupings that were assumed to have unequal variances in the linear mixed model.

**Details**

This function provides the necessary steps to apply the qusage algorithm to differential gene expression analysis that were conducted using more statistically advanced models. Only the original qusage package is necessary to be installed to run this function.

Note that when random effects are present, it is still up to the user to determine if the residuals matrix provided for the analysis is the conditional residual matrix or a marginal residual matrix treating the random effects as fixed effects. For large number of random effect replicates, it will not make much difference as the two approaches will converge, however for a small number of replicates, say 2 to 5, we recommend the latter approach in providing the residual matrix. This decision is solely for the purpose of VIF estimation, the t-statistic information should be given based on the model using fixed and random effects as they were originally specified.
